# Model-Informed Precision Dosing Software Tools for Dosage Regimen Individualization: A Scoping Review

**DOI:** 10.3390/pharmaceutics15071859

**Published:** 2023-07-01

**Authors:** Paula Del Valle-Moreno, Paloma Suarez-Casillas, Marta Mejías-Trueba, Pablo Ciudad-Gutiérrez, Ana Belén Guisado-Gil, María Victoria Gil-Navarro, Laura Herrera-Hidalgo

**Affiliations:** 1 Department of Pharmacy, University Hospital Virgen del Rocío, 41013 Seville, Spain; pauladelvallem.08@hotmail.com (P.D.V.-M.); palomasuacas@gmail.com (P.S.-C.); pablocg95@gmail.com (P.C.-G.); anaguigil@gmail.com (A.B.G.-G.); mariav.gil.sspa@juntadeandalucia.es (M.V.G.-N.); lauraherrerahidalgo@gmail.com (L.H.-H.); 2 Department of Infectious Diseases, Microbiology and Parasitology, Infectious Diseases Research Group, Institute of Biomedicine of Seville, University of Seville/Spanish National Research Council/University Hospital Virgen del Rocio, 41013 Seville, Spain; 3 Centre for Biomedical Research Network on Infectious Diseases, 28029 Madrid, Spain

**Keywords:** model-informed precision dosing, software tool, therapeutic drug monitoring

## Abstract

Background: Pharmacokinetic nomograms, equations, and software are considered the main tools available for Therapeutic Drug Monitoring (TDM). Model-informed precision dosing (MIPD) is an advanced discipline of TDM that allows dose individualization, and requires a software for knowledge integration and statistical calculations. Due to its precision and extensive applicability, the use of these software is widespread in clinical practice. However, the currently available evidence on these tools remains scarce. Objectives: To review and summarize the available evidence on MIPD software tools to facilitate its identification, evaluation, and selection by users. Methods: An electronic literature search was conducted in MEDLINE, EMBASE, OpenAIRE, and BASE before July 2022. The PRISMA-ScR was applied. The main inclusion criteria were studies focused on developing software for use in clinical practice, research, or modelling. Results: Twenty-eight software were classified as MIPD software. Ten are currently unavailable. The remaining 18 software were described in depth. It is noteworthy that all MIPD software used Bayesian statistical methods to estimate drug exposure and all provided a population model by default, except NONMEN. Conclusions: Pharmacokinetic software have become relevant tools for TDM. MIPD software have been compared, facilitating its selection for use in clinical practice. However, it would be interesting to standardize the quality and validate the software tools.

## 1. Introduction

Clinical pharmacokinetics is a field dedicated to optimizing the dosing regimens of specific medications, aiming to enhance their effectiveness while minimizing undesirable effects in clinical practice [1,2]. In practical terms, therapeutic drug monitoring (TDM) serves as the application of this discipline in a clinical setting, involving individualized dosing based on tracking drug concentrations. By utilizing this information, dose optimization can be achieved through an assessment of concentration exposure [3]. Primary tools employed for TDM include pharmacokinetic (PK) nomograms, equations, and software. Unfortunately, therapeutic failures and toxicities remain prevalent, particularly in medications and populations exhibiting substantial inter- and intraindividual PK variability [1,2].

To address this issue, computer programs have been developed to assist professionals in predicting the optimal dosage regimen for individual patients [1,2]. Model-informed precision dosing (MIPD) represents an advanced discipline within TDM, providing dose individualization primarily based on TDM measurements, and secondarily on PK population models that account for individual characteristics and intra/inter-patient variabilities. The achievement of this approach necessitates the integration of knowledge and mathematical optimization within specialized software [4].

Various software programs have been developed to facilitate predictions of patient drug concentrations and provide dose recommendations that account for individual variations within the population model. These predictions typically rely on the Bayesian theorem [5,6]. The increasing use of MIPD software can be attributed to its precision, the advancement of population pharmacokinetic models, and the expanding range of drugs that can be optimized. Additionally, there is a growing recognition of the importance of dose individualization for vulnerable populations, including elderly patients, those with renal or hepatic insufficiency, pregnant women, critically ill patients, and pediatric patients. Consequently, computer programs have become highly valued tool sin routine clinical practice [7,8].

However, the widespread adoption of MIPD software is hindered by limited accessibility and a lack of comprehensive information about their features and clinical applicability (among other reasons). This lack of information hampers efficient and effective comparative analysis among different software options. Moreover, some authors argue that most pharmacokinetic monitoring software requires further development to enhance user-friendliness, data storage capacity, and report generation [6]. Consequently, it is essential to evaluate the current state of software tools.

The main objective of this scoping review is to identify software specifically designed to support MIPD and provide a comprehensive description of their main features. Special emphasis is given to software intended for use in clinical settings, aiming to assist healthcare professionals in selecting the most suitable software tool to meet their specific needs.

## 2. Methods

### 2.1. Protocol and Registration

A scoping review was performed in accordance with PRISMA-ScR (Preferred Reporting Items for Systematic reviews and Meta-Analyses extension for Scoping Reviews) guidelines [9], and following the protocol published previously [10]. The review was registered on the international registry Open Science Framework (ID: 10.17605/OSF.IO/M53NF).

### 2.2. Data Source and Search Strategy

A literature search was performed in MEDLINE (PubMed) and EMBASE using controlled vocabulary and covering the literature published until 31 July 2022. A complementary search was also conducted in the OpenAIRE and BASE (Bielefeld Academic Search Engine) electronic databases to consult the information published as grey literature. In order to conduct the search and minimize potential publication bias, all the references of the articles found were carefully examined, which made it possible to identify studies that had not been detected during the review. The detailed search strategy is shown in the Supplementary Material (Table S1).

### 2.3. Articles Eligibility Criteria

#### 2.3.1. Inclusion Criteria

- Studies focused on the design or development of MIPD software tools, as well as studies that provide detailed descriptions on the features of software tools available for use in pharmacokinetics.
- Studies including software tools aimed at any medication class.

#### 2.3.2. Exclusion Criteria

- Studies focused on applying an existing software in a patient cohort for TDM without explaining the origin of the development of the software.
- Studies that include software that is only available in a particular country.
- Articles in a language other than English or Spanish.
- Articles not available in full text.

### 2.4. Study Screening and Selection

Firstly, duplicate articles were eliminated. Thereafter, two reviewers (PVM and PCG) independently selected the articles using the aforementioned inclusion criteria, based on the information obtained from the title and abstract. When in doubt, they read the entire article before deciding whether to include it. Next, the full texts of the articles selected for the reviewers were read and those who met the eligibility criteria were selected. To ensure reproducibility and minimal bias, discrepancies were resolved through discussion and consensus with other reviewers (MMT and LHH). The reasons for the studies’ exclusion were recorded.

### 2.5. Software Selection and Data Collection

Software designed or suitable for MIPD in the clinical setting were selected and fully described. The software designed for research or for population pharmacokinetic modelling were identified and presented in a table.

Reviewer PVM independently extracted data, and PSC and MMT examined all extraction sheets to ensure their accuracy. When the software information provided by the studies was insufficient to make an appropriate selection, we conducted an additional search on digital platforms or contacted application developers to request information and guarantee the software met the characteristics described.

The collected variables were classified into different categories:

(a) Focused on the characteristics of the articles included: year and country of publication, aim of studies, and software described.
(b) Focused on the characteristics of the identified software:
  - General characteristics:
    1. Developer/promoter: the name of the authors, institution, or company participating in software performance.
    2. Year of creation: the year when the first version of the software was created.
    3. Geographical location: the country where the software was developed.
    4. Languages in which the software is available.
    5. Last update of the software.
    6. Use: MIPD, research, or population pharmacokinetic modelling.
  - Access options:
    1. Possibility of testing the software before contracting it.
    2. Subscription: paid or free access.
    3. Platforms: desktop, web-based, or mobile application.
  - Technical characteristics:
    1. Pharmacokinetic analysis type available: Bayesian or non-Bayesian.
    2. Inclusion or not of population data by default: provision or not of a population model created by the software developers.
    3. Possibility of including new drugs and populations by the user or upon request to the developer.
    4. Possibility of issuing reports and creating graphs.
    5. Integration with other systems: Electronic Health Records/Electronic Medical Records (EHR/EMR).
  - Potential clinical applicability:
    1. Group of drugs classified according to Anatomic Therapeutic Chemical (ATC) code included in the software.
    2. Target population of the models included in the software.
    3. User-friendliness was independely evaluated by two researchers (PVM and LHH).

## 3. Results

### 3.1. Literature Search

A total of 1950 articles were identified in the databases searched (836 in PubMed, 565 in EMBASE, 514 in OpenAIRE, and 35 in BASE) and 4 papers were included after reviewing the literature references. After removing duplicates, a total of 1643 articles remained. Among these, 1227 articles were excluded based on the title and abstract review.

The remaining 416 studies were considered potentially relevant, and their full texts were retrieved for further examination. From this subset, 391 were excluded before data extraction and 25 met the inclusion criteria (Supplementary Figure S1).

### 3.2. Characteristics of the Articles and Software

Among the 25 articles included (Supplementary Table S2), 37 software tools were identified.

The 37 software tools identified were classified: 8 population modelling software, 1 research software, and 28 MIPD software. Additionally, four software were located by manual search: ADAPT, iDose [11], RxStudio [12], and CAPCIL. The last two programs mentioned are the new versions of ID-ODS and SIMKIN, respectively. Supplementary Table S3 shows the software identified and designed for modelling or research.

During the analysis, a total of 28 MIPD software were thoroughly examined. However, 10 of these software were excluded for various reasons. Five of them were no longer available or marketed (PHAR-MONITOR, PKRD, OPT, DataKineticsTM, and RADKi-netics). Four software were previous versions of other included software, and the newer versions were considered instead (SIMKIN, USC*PACK, T.D.M.S. 2000, and ID-ODS). TDM for R was dismissed as it is a JPKD plugin.

A full description of the main characteristics of the 18 MIPD software selected are detailed in Table 1. These software were developed between the years 1979 and 2020. Nine of them were developed by commercial companies, while the remaining were created by noncompany providers. Seven software have been updated within the last year. Eleven software offer the option of a free trial, except for NextDose, which does not provide this option. Information regarding free access was not available for the remaining six software. Regarding free access, it is available for four software (JPKD, TCIWorks, TUCUXI, and TDMx). RxStudio and NextDose offer different types of licenses, including paid and free subscriptions with varying functionalities. For instance, the free access to RxStudio only includes simulations of empiric treatments. The access method for CAPCIL is unknown.

Out of the 18 selected MIPD software, 12 of them are web-based. Among these, four software (Autokinetics, MwPharm, PrecisePK, and RxStudio) also offer a desktop version in addition to the web-based platform. Additionally, DoseMeRx, MwPharm, and RxStudio can also be accessed through mobile applications. The remaining software are solely available in desktop versions.

All of the MIPD software analyzed in the study employ Bayesian statistical methods to estimate drug exposure and generate dosing recommendations. From a statistical perspective, population PK modelling approaches can be categorized as either parametric or nonparametric [13]. In the parametric Bayesian approach, the population parameters are treated as random variables with known prior distributions. Estimating the conditional distribution of the population parameters can be challenging in this case. On the other hand, the nonparametric approach assumes that the population distribution is completely unknown and random, making it a more flexible Bayesian approach [13]. It is worth noting that Autokinetics, CAPCIL, and PKS additionally offer the capability to perform linear and nonlinear regression.

**Table 1:** Descriptive characteristics of the MIPD software.

	**Autokinetics**	**BESTDOSE**	**CAPCIL**	**DoseMeRx**	**iDose**	**InsightRX Nova**	**JPKD**	**Kinetidex**	**MwPharm**
**General characteristics**									
Developer/ promoter	Paul Elbers, Rob Bosman. Departments of Intensive Care Medicine of Amsterdam UMC	R.W. Jelliffe. Laboratory of Applied Pharmaco-kinetics, University of Southern California, LA. Non-company owners	Company (SIMKIN Inc.)	Robert McLeay. DoseMeRx^®^ (Tabula Rasa Healthcare Company)	Company (Projections Research Inc. Baysient^®^)	Sirj Goswami, Ron Keizer and Ranvir Mangat. Company (Insight Rx Inc.)	College of Pharmacy, Kaohsiung Medical University (Taiwan)	Thomson Reuters corp. (merge between Simkin and Micromedex)	Johannes H. Proost Department of Pharmacology and Therapeutics, University Centre for Pharmacy, Groningen. Company (Mediware a.s.)
Year of creation	2018	2018	N/A	2014	N/A	2015	2006	2001	1987
Geographical location	Netherlands	USA	USA	USA	USA	USA	Taiwan	USA	Netherlands
Languages available	English	English *	N/A	English	English	English, Dutch, German, French. Possibility of development	English *	English	English, Spanish, Dutch, German, Korean, Czech, Portuguese, Japanese, Chinese
Last update	N/A	Version 2.4.3 Previous version 1973: MM-USC*Pack [7]	N/A	N/A	November 2022	The platform is updated regularly (once a month). Previous version: InsightRX software [14]	2008 version 3.0	2009 Version 9.0	September 2022 Version 2.2.0
**Access options**									
Testing the software	Yes	Yes	N/A	Yes, a 14-day trial can be generated	Yes	Yes	N/A	N/A	Yes
Subscription	Paid access	Paid access	Unknown	Paid access	Paid access	Paid access	Free access	Paid access	Paid access
Platforms	Desktop and web-based: www.autokinetics.eu	Web-based www.lapk.org/bestdose.php	Desktop	Mobile application and web-based: https://doseme-rx.com/	Web-based: www.baysient.net/idose-product/	Web-based: www.insight-rx.com/	Web-based: pkpd.kmu.edu.tw/jpkd/	Desktop	Desktop, mobile application and web-based: www.mediware.cz/
**Technical characteristics**									
Pharmacokinetic analysis type available	Bayesian and non-bayesian	Bayesian (non-parametric approach)	Bayesian and non-bayesian	Bayesian (parametric approach)	Bayesian	Bayesian	Bayesian	Bayesian	Bayesian (parametric approach)
Inclusion/not by default of population data	Yes	Yes, it offers a collection of population PK models	N/A	Yes, it offers a collection of population PK models	Yes	Yes, it offers a collection of population PK models.	N/A	Yes	Yes, it offers a collection of population PK models
Inclusion of drugs and populations PK models	No	No	N/A	Yes, upon request.	Yes, upon request.	Yes, upon request.	Yes, by the user.	No	Yes, by the user or upon request.
Issuance of reports, creation of graphs	Reports cannot be generated. Graphical representation can be generated (not exported).	Reports cannot be generated.	Reports and graphical representation can be generated.	Reports and graphical representation can be generated.	Graphical representation can be generated.	Reports and graphical representation can be generated.	Reports can be generated.	Reports and graphical representation can be generated.	Reports and graphical representation can be generated.
Integration into EHR/EMR	Yes. It can be integrated	N/A	N/A	Yes. It can be integrated	N/A	Yes. It can be integrated	N/A	N/A	Yes. It can be integrated
**Clinical applicability**									
Drug class	Antibiotics	Antibiotics, digoxin	Antibiotics, digoxin, lidocaine, quinidine, theophylline	Antibiotics, anticoagulants, anticonvulsants antifungals, antineoplastics, antithrombotic, immunosuppressants (drugs for transplants), digoxin, warfarin	Immunosuppressants (biological agents)	Antibiotics, anticoagulants (oral, factors), antifungals, antineoplastics, antipsychotics, antithrombotic, digoxin, immunosuppressants (both drugs for transplants and biological agents), methadone	Anticonvulsant, antiepileptic, antivirals, digoxin, immunosuppressants (drugs for transplants), lithium, theophylline, warfarin	Antibiotics, antiepileptic, digoxin, theophylline, warfarin	Antibiotics, antiepileptics, antihypertensives, antivirals, immunosuppressants (biological agents), digoxin, warfarin
Target population †	N/A	N/A	Adults, neonates and paediatrics	Adults, neonates and paediatrics. Haemodialysis and obese	Adults and paediatrics	Adults, neonates and paediatrics	N/A	N/A	Adults, neonates and paediatrics. Critically ill patients and haemodialysis
	**NextDose**	**NONMEM**	**PrecisePK**	**PKS**	**RxKinetics**	**RxStudio**	**TCIWorks**	**TUCUXI**	**TDMx**
**General characteristics**									
Developer/ promoter	Sam Holford Nick Holford. University of Auckland Non-company owned	Project Group at the University of California, San Francisco Company (NONMEM^®^)	Philip Anderson, Anjum Gupta. Company (Healthware Inc.)	Company (Abbott Laboratories, Diagnostic)	School of Pharmacy and Health Profession, Creighton University	Ajay Gopal, Gergely Daroczi. Company (Rx Studio Inc.)	University of Queensland (Australia) and University of Otago (New Zealand)	Yann Thoma. School of management and engineering of Vaud and the University Hospital of Lausanne	Sebastian Wicha. Institute of Pharmacy, University of Hamburg
Year of creation	2012	1979	1986	1991	1984	2020	2011	2013	2015
Geographical location	New Zealand	USA	USA	USA	USA	USA/Hungary	New Zealand and Australia	Switzerland	Germany
Languages available	English	English	English, Spanish, Korean, Chinese, Thai	English	English	English, Spanish, Portuguese, French, Hungarian, and Simplified Chinese	English *	English	German, English
Last update	September 2022	NONMEM 7.5.1 February 2022	September 2022 Previous version: T.D.M.S.	N/A	November 2021	February 2023. The platform is updated regularly. Previous version: ID-ODS	N/A	November 2022	February 2023
**Access options**									
Testing the software	No	N/A	Yes, a 30-day trial can be generated	N/A	Yes, a 60-day trial version can be downloaded	Yes	N/A	Yes	Yes
Subscription	Paid or free access	Paid access	Paid access	Paid access	Paid access	Paid access. Free individual access for empirical simulations.	Free access	Free access	Free access
Platforms	Web-based: nextdose.org/	Desktop	Desktop and web-based: precisepk.com/	Desktop	Web-based: www.rxkinetics.com/	Desktop, mobile application, web-based: rx.studio/	Desktop	Desktop	Web-based: www.tdmx.eu/
**Technical characteristics**									
Pharmacokinetic analysis type available	Bayesian	Bayesian	Bayesian	Bayesian and non-bayesian	Bayesian	Bayesian (parametric approach)	Bayesian (parametric approach)	Bayesian	Bayesian
Inclusion/not by default of population data	Yes, it offers a collection of population PK models	No. User may define model for any drug or target population	Yes, it offers a collection of population PK models	Yes, by the user	Yes	Yes, it offers a collection of population PK models	Yes	Yes, it offers a collection of population PK models	Yes, it offers a collection of population PK models
Inclusion of drugs and populations PK models	No	Yes, by the user.	Yes, upon request.	Yes, generation of new drugs in different populations	Yes, upon request.	Yes, upon request.	Yes, by the user.	Yes, by the user and supported by the developer.	Only new population (not new drugs), implemented by the user.
Issuance of reports, creation of graphs	Reports and graphical representation can be generated	Reports can be generated	Reports can be generated	Reports and graphical representation can be generated	Reports and graphical representation can be generated	Reports and graphical representation can be generated	Reports can be generated	Reports and graphical representation can be generated.	Reports cannot be generated. Graphical representation can be generated (not exported)
Integration into EHR/EMR	No	N/A	Yes. It can be integrated	N/A	N/A	Yes. It can be integrated	N/A	Yes. It can be integrated	No
**Clinical applicability**									
Drug class	Antibiotics, anticoagulants, antifungals, antineoplastics, antirheumatics, antivirals, immunosuppressants (drugs for transplants), psychostimulant, warfarin	N/A (selected by the user)	Antiarrhythmicantiasthmatics, antibiotics, antiepileptics, antifungals, antineoplastics, antipsychotics, immunosuppressants (drugs for transplants)	Antibiotics, digoxin, methotrexate, phenytoin, theophylline	Antibiotics, digoxin	Antibiotics, antifungals, anticonvulsants, biological agents, immunosuppressants (drugs for transplants), methotrexate, opioids	Antibiotics, immunosuppressants (drugs for transplants), theophylline, warfarin	Antibiotics, anticoagulants, antivirals, antineoplastics, immunosuppressants (drugs for transplants), kinase inhibitors	Antibiotics, haemostatics (factors), immunosuppressants (biological agents)
Target population †	Adults, neonates and paediatrics	N/A	Adults, neonates, paediatrics. Critically ill patients, obese, renal impairment and haemodialysis	Adults and paediatrics. Critically ill patients	Adults and paediatrics	Adults, neonates and paediatrics. Haemodialysis and haematology patients	Adults and paediatrics	Adults, neonates and paediatrics	Adults, neonates and paediatrics

* Availability in other languages unknown. † At least one model per target population.

By default, all the MIPD software provide a population model, except for NONMEN, within which models might be defined by users. Thirteen software include models developed for both adult and pediatric populations. Nine software also include models specific to the neonatal population. Moreover, several software tools offer models for special populations: dialysis patients (DoseMeRx, MwPharm, PrecisePK, and RxStudio), obese patients (DoseMeRx and PrecisePK) and critically ill patients (MwPharm, PrecisePK, and PKS). Information regarding this aspect was not available for Autokinetics, BestDose, JPKD, and Kinetidex.

Among the MIPD software analyzed, there are options for including new population PK models and drugs based on user requests or developer support. Seven software (DoseMeRx, iDose, InsightRX Nova, MwPharm, PrecisePK, RxKinetics, and RxStudio) allow the inclusion of new population PK models and drugs upon user request, while four software (JPKD, MwPharm, NONMEM, and TCIWorks) enable users to add their own models. TUCUXI allows users to include new population PK models and drugs, supported by the developer. TDMx offers the possibility of user-defined new population models but only for the drugs already included by default in the software. InsightRX Nova provides automated population model selection based on patient input data.

Fifteen MIPD software include the option to generate reports, and ten of them support graphical representation. Integration with EHR is feasible with seven software (Autokinetics, DoseMeRx, InsightRX Nova, MwPharm, PrecisePK, RxStudio, and TUCUXI).

Regarding the preset drugs accessible in each software, antibiotics are the most commonly implemented drug class. Immunosuppressants are included in eleven software, with specific drugs used in transplant patients such as mTOR inhibitors being included in DoseMeRx, JPKD, NextDose, PrecisePK, RxStudio, TCIWorks, and TUCUXI. Biologic immunomodulator agents are included in iDose, MwPharm, and TDMx. InsightRX Nova covers both transplant drugs and biologics. Antiepileptics are incorporated in four software (JPKD, Kinetidex, MwPharm, and PrecisePK) while kinase inhibitors are only included in TUCUXI. InsightRX Nova covers the most extensive range of drug classes, while Autokinetics and iDose focus on antibiotics and biological agents, respectively. NONMEN does not include any preset drugs, and all drugs must be added by the user.

Ease-of-use was assessed based on factors such as simple access, a user-friendly interface, and a smooth flow through the necessary steps to perform a simulation. Among the 18 included software, seven were considered intuitive and user-friendly (Tucuxi, PrecisePK, RxStudio, MwPharm, iDose, DoseMe, and InsightRX Nova). All of these software were web-based and had the capability of EHR integration, which enhanced the user experience. They also shared a modern interface where the path to run a simulation was easily identifiable. Four other software (NextDose, BestDose, TDMx, and RxKinetics) were also considered relatively easy to use, although their interfaces were less user-friendly and the workflow was less assisted compared to the previous group. PKS and JPKD were not considered user-friendly due to their outdated interfaces and lack of smooth flow through the screens. NONMEN deserves a special mention as its use requires a high level of expertise not only in pharmacokinetics and pharmacometrics but also in programming, making it less accessible for clinical settings. Lastly, Kinetidex, Autokinetics, CAPCIL, and TCIworks were not accessible to the investigators, so their ease-of-use could not be evaluated.

## 4. Discussion

This manuscript serves as a scoping review aimed at identifying the MIPD software available globally and providing a comprehensive description of their main features. The objective is to facilitate the selection of the most suitable software for health care professionals in clinical practice.

Selecting the ideal MIPD software tool can be challenging due to the wide range of available programs and limited accessible information. The lack of information about certain software hinders its potential use in clinical practice, rendering it ineffective. Additionally, locating web-based access to multiple software platforms can be a difficult task.

Previous nonsystematic studies have explored MIPD software and provided valuable information in the field [1,7,14,15]. Buffington et al. conducted a narrative review in 1993, aiming to compare pharmacokinetic software programs and assist clinicians in selecting the most suitable software for their clinical settings [15]. They analyzed thirteen software tools, five of which were included in the present scoping review in their updated versions. The remaining eight software tools are no longer commercially available. In 2013, a benchmarking study was conducted [7], identifying and analyzing twelve software tools for TDM clinical activities. While all the software mentioned in that study were also included in the present review, two of them (DataKinetics and RADKi-netics) were excluded as they are not currently available. The updated versions of the remaining software tools are described in the present study. A more recent narrative review [14] focused on four commercially available MIPD software tools that enable clinicians to apply PK-PD principles to optimize dosing regimens (DoseMeRx, InsightRX, PK-PD Compass, and TDMx). However, the literature search conducted in that study was limited and not systematic, and no benchmarking analysis was performed. Another comparative study in 2020 evaluated the main features and performance of ten MIPD software tools available at that time [1]. The results of this study differ from those proposed in 2013 and in the present review, due to the frequent updates and obsolescence of software tools. Having up-to-date information is crucial for selecting the most appropriate software.

Indeed, comparing the MIPD software tools included in Buffington et al.’s study [15] with the findings of our scoping review highlights the advancements and improvements that have occurred over the years. PrecisePK, formerly known as TDMS, has evolved to become one of the most complete software tools. It now includes features such as a population PK model, report generation capability, and EHR integration, and a wide range of drug classes have been implemented. The ease of manual data entry with PrecisePK was also noted in Kantasiripitak et al. [1]. Similarly, USC*PACK, which was recognized as one of the earliest and most widely used clinical pharmacokinetic programs in 1993 [15], has undergone transformations and is now represented by the software tool BestDose. Although BestDose may not have ranked among the most attractive software tools based on our analysis, it continues to be utilized in clinical practice.

One notable difference between the previous studies and our scoping review is the shift towards web-based software tools. The majority of the software tools included in our review, as well as in the recent narrative review, are web-based, offering greater accessibility and ease-of-use. This transition to web-based platforms, coupled with regular updates, reflects the trend towards more sophisticated, user-friendly, and intuitive MIPD software tools that are increasingly utilized by clinicians.

The implementation of MIPD software has indeed brough about significant advancements in individualized dosage optimization, benefiting both research and clinical practice. These tools have the potential to revolutionize precision dosing by addressing the considerable inter- and intraindividual variability in patient populations, leading to more effective and safer treatment outcomes. In the hospital setting, where patients often have complex conditions and exhibit significant PK alterations, the importance of MIPD software becomes even more pronounced [16]. The ability to individualize dosage regimens for a wide range of drugs is crucial in optimizing patient care and achieving desirable therapeutic outcomes. To be effective and widely adopted, MIPD software should possess certain key characteristics. They should be user-friendly, offering simplicity and flexibility in dosage individualization. This means providing the user with the tools and features necessary to tailor doses in various clinical situations, accommodating both simple and complex patient populations. Additionally, ideal software should have data store capability and EHR integration [1,7,16].

The evaluation of ease-of-use and EHR integration in MIPD software has been a focus of previous studies, providing valuable insights into these important aspects. Fuchs et al. [7] emphasized the need for MIPD software tools to evolve towards comprehensive tools with clinical and research capabilities, while maintaining user-friendliness. In their evaluation, MwPharm, MM-USC*PACK (currently BestDose), and TCIWorks were identified as the most sophisticated programs. Similarly, Kantasiripitak et al. [1] conducted a user-friendliness evaluation and highlighted DoseMeRx, InsightRX Nova, and MwPharm as meeting the requirements. These software tools were also found to be compatible with her integration. These findings align with our study, where we identified seven out of the eighteen MIPD software as being prepared for EHR integration (Autokinetics, DoseMeRx, InsightRX Nova, MwPharm, PrecisePK, RxStudio, and TUCUXI), making them particularly appealing. The perception of ease-of-use is subjective and influenced by the expertise of the reviewer. However, the agreement between our study´s evaluation and previous findings, particularly regarding the intuitiveness and user-friendliness of web-based software prepared for EHR integration, suggests a consistent understanding of these important features.

Indeed, the quality of the software, user support services, supported drugs and population models, as well as output generation, are crucial considerations for selecting a MIPD software. The study by Kantasiripitak et al. [1] positioned DoseMeRx, InsightRX Nova, and MwPharm as excellent options for dose optimization in clinical practice. Similarly, the review by Fuchs et al. [7] identified MwPharm, MM-USC*PACK (currently BestDose), and TCIWorks as the most complete programs. In our opinion, software that includes population PK models for special populations, such as obese patients, critically ill patients, neonates, or patients on dialysis, provides added benefits for professionals who frequently treat patients with more complex requirements. PrecisePK stands out as the most complete tool in this regard, as it includes models for each of the mentioned target populations. Additionally, DoseMeRx, MwPharm, and RxStudio offer the ability to predict optimal dosing regimens for various special populations. Apart from special populations, DoseMeRx, InsightRX Nova, and MwPharm provide the largest collection of population PK models by default. These aspects facilitate the implementation of such software in clinical practice.

The study acknowledges several limitations that should be taken into consideration. Firstly, the usability of each program was not directly assessed, and there was no testing with real clinical cases of precision dosing. This limitation was due to the potential time constraints of the study. Secondly, feedback from some software developers could not be obtained, which may have affected the completeness of the data. Thirdly, despite carrying out a systematic and well-designed search strategy across numerous databases, consulting grey literature and using MESH terms, it is possible that not all currently available MIPD software tools were detected. This fact reveals the existence of another barrier, which hinders access to software and the application of TDM/MIPD in clinical practice. Additionally, the study did not include a comparison of the accuracy of each program.

Based on the findings, the authors suggest that information on MIPD software tools should be more accessible to healthcare professionals to facilitate their implementation and promote greater integration into clinical practice. Quality standardization and validation of software tools would be interesting for more accurate selection. Based on the extensive information gathered, DoseMeRx, InsightRx Nova, MwPharm, PrecisePK, and RxStudio are identified as potentially accurate and suitable optimizing dosing regimens in clinical practice. Nevertheless, it is emphasized that software tools should be tested with real patient situations to assess their performance in terms of population and individual patient PK characteristics, accuracy of predictions, and other aspects such as intuitiveness or user-friendliness.

In conclusion, this scoping review serves as a valuable resource for healthcare professionals by providing relevant information to aid in the selection of MIPD software tools available for routine clinical practice. The review emphasizes the importance of accessibility and availability of information on MIPD software tools to promote their implementation in clinical practice and calls for further standardization, validation, and real-world testing to assess the performance and accuracy of these tools.

## Data Availability

All data is published in the article or as supplementary material.

## Supplementary Materials

The following supporting information can be downloaded at: https://www.mdpi.com/article/10.3390/pharmaceutics15071859/s1, Figure S1. Preferred Reporting Items for Systematic reviews and Meta-Analyses extension for Scoping Reviews (PRISMA-ScR) Checklist [9]. Table S1. Search strategy. Table S2. Summary of the articles included in this review. Table S3. Software tools for modelling or research. References [14,17,18,19,20,21,22,23,24,25,26,27,28,29,30,31,32,33,34,35,36] are cited in the supplementary materials.
