# Correction: Del Valle-Moreno et al. Model-Informed Precision Dosing Software Tools for Dosage Regimen Individualization: A Scoping Review. *Pharmaceutics* 2023, *15*, 1859

**DOI:** 10.3390/pharmaceutics16060766

**Published:** 2024-06-04

**Authors:** Paula Del Valle-Moreno, Paloma Suarez-Casillas, Marta Mejías-Trueba, Pablo Ciudad-Gutiérrez, Ana Belén Guisado-Gil, María Victoria Gil-Navarro, Laura Herrera-Hidalgo

**Affiliations:** 1 Department of Pharmacy, University Hospital Virgen del Rocío, 41013 Seville, Spain; pauladelvallem.08@hotmail.com (P.D.V.-M.); palomasuacas@gmail.com (P.S.-C.); pablocg95@gmail.com (P.C.-G.); anaguigil@gmail.com (A.B.G.-G.); mariav.gil.sspa@juntadeandalucia.es (M.V.G.-N.); lauraherrerahidalgo@gmail.com (L.H.-H.); 2 Department of Infectious Diseases, Microbiology and Parasitology, Infectious Diseases Research Group, Institute of Biomedicine of Seville, University of Seville/Spanish National Research Council/University Hospital Virgen del Rocio, 41013 Seville, Spain; 3 Centre for Biomedical Research Network on Infectious Diseases, 28029 Madrid, Spain

There was an error in the original publication [1]; there is a mistake in the number of software. A correction has been made in the Abstract:

“Background: Pharmacokinetic nomograms, equations, and software are considered the main tools available for Therapeutic Drug Monitoring (TDM). Model-informed precision dosing (MIPD) is an advanced discipline of TDM that allows dose individualization, and requires a software for knowledge integration and statistical calculations. Due to its precision and extensive applicability, the use of these software is widespread in clinical practice. However, the currently available evidence on these tools remains scarce. Objectives: To review and summarize the available evidence on MIPD software tools to facilitate its identification, evaluation, and selection by users. Methods: An electronic literature search was conducted in MEDLINE, EMBASE, OpenAIRE, and BASE before July 2022. The PRISMA-ScR was applied. The main inclusion criteria were studies focused on developing software for use in clinical practice, research, or modelling. Results: Twenty-eight software were classified as MIPD software. Nine are currently unavailable. The remaining 19 software were described in depth. It is noteworthy that all MIPD software used Bayesian statistical methods to estimate drug exposure and all provided a population model by default, except NONMEN. Conclusions: Pharmacokinetic software have become relevant tools for TDM. MIPD software have been compared, facilitating its selection for use in clinical practice. However, it would be interesting to standardize the quality and validate the software tools.”

The corrections have been made to *Section 3.2. Characteristics of the Articles and Software* in order to include ID-ODS software, which was originally mistaken as an old version of RxStudio: 

“Among the 25 articles included (Supplementary Table S2), 37 software tools were identified.

The 37 software tools identified were classified: 8 population modeling software, 1 research software, and 28 MIPD software. Additionally, four software were located by manual search: ADAPT, iDose [11], RxStudio [12], and CAPCIL. The last program mentioned is the new version of SIMKIN. Supplementary Table S3 shows the software identified and designed for modeling or research.

During the analysis, a total of 28 MIPD software were thoroughly examined. However, nine of these software were excluded for various reasons. Five of them were no longer available or marketed (PHAR-MONITOR, PKRD, OPT, DataKineticsTM, and RADKi-netics). Three software were previous versions of other included software, and the newer versions were considered instead (SIMKIN, USC*PACK, and T.D.M.S. 2000). TDM for R was dismissed as it is a JPKD plugin.

A full description of the main characteristics of the 19 MIPD software selected are detailed in Table 1. These software were developed between the years 1979 and 2020. Ten of them were developed by commercial companies, while the remaining were created by non-company providers. Eight software have been updated within the last year. Twelve software offer the option of a free trial, except for NextDose, which does not provide this option. Information regarding free access was not available for the remaining six software. Regarding free access, it is available for four software (JPKD, TCIWorks, TUCUXI, and TDMx). RxStudio, ID-ODS, and NextDose offer different types of licenses, including paid and free subscriptions with varying functionalities. For instance, the free access to RxStudio only includes simulations of empiric treatments. The access method for CAPCIL is unknown.

Out of the 19 selected MIPD software, 13 of them are web-based. Among these, four software (Autokinetics, MwPharm, PrecisePK, and RxStudio) also offer a desktop version in addition to the web-based platform. Additionally, DoseMeRx, MwPharm, and RxStudio can also be accessed through mobile applications. The remaining software are solely available in desktop versions.

All of the MIPD software analyzed in the study employ Bayesian statistical methods to estimate drug exposure and generate dosing recommendations. From a statistical perspective, population PK modeling approaches can be categorized as either parametric or nonparametric [13]. In the parametric Bayesian approach, the population parameters are treated as random variables with known prior distributions. Estimating the conditional distribution of the population parameters can be challenging in this case. On the other hand, the nonparametric approach assumes that the population distribution is completely unknown and random, making it a more flexible Bayesian approach [13]. It is worth noting that Autokinetics, CAPCIL, and PKS additionally offer the capability to perform linear and nonlinear regression.

By default, all the MIPD software provide a population model, except for NONMEN, within which models might be defined by users. Fourteen software include models developed for both adult and pediatric populations. Ten software also include models specific to the neonatal population. Moreover, several software tools offer models for special populations: dialysis patients (DoseMeRx, MwPharm, PrecisePK, ID-ODS, and RxStudio), obese patients (ID-ODS, DoseMeRx, and PrecisePK), and critically ill patients (MwPharm, ID-ODS, PrecisePK, and PKS). Information regarding this aspect was not available for Autokinetics, BestDose, JPKD, and Kinetidex.

Among the MIPD software analyzed, there are options for including new population PK models and drugs based on user requests or developer support. Eight software (DoseMeRx, ID-ODS, iDose, InsightRX Nova, MwPharm, PrecisePK, RxKinetics, and RxStudio) allow the inclusion of new population PK models and drugs upon user request, while four software (JPKD, MwPharm, NONMEM, and TCIWorks) enable users to add their own models. TUCUXI allows users to include new population PK models and drugs supported by the developer. TDMx offers the possibility of user-defined new population models but only for the drugs already included by default in the software. InsightRX Nova provides automated population model selection based on patient input data. ID-ODS includes free-style simulation routines that allow the incorporation of one-, two-, and three-compartment models for the drugs available in the system.

Sixteen MIPD software include the option to generate reports, and eleven of them support graphical representation. Integration with EHR is feasible with seven software (Autokinetics, DoseMeRx, InsightRX Nova, MwPharm, ID-ODS, PrecisePK, RxStudio, and TUCUXI).

Regarding the preset drugs accessible in each software, antibiotics are the most commonly implemented drug class. Immunosuppressants are included in eleven software, with specific drugs used in transplant patients, such as mTOR inhibitors, being included in DoseMeRx, JPKD, NextDose, PrecisePK, RxStudio, TCIWorks, and TUCUXI. Biologic immunomodulator agents are included in iDose, MwPharm, and TDMx. InsightRX Nova covers both transplant drugs and biologics. Antiepileptics are incorporated in four software (JPKD, Kinetidex, MwPharm, and PrecisePK), while kinase inhibitors are only included in TUCUXI. InsightRX Nova covers the most extensive range of drug classes, while Autokinetics and iDose focus on antibiotics and biological agents, respectively. ID-ODS focuses on antibiotics and antifungal agents. NONMEN does not include any preset drugs, and all drugs must be added by the user.

Ease of use was assessed based on factors such as simple access, a user-friendly interface, and a smooth flow through the necessary steps to perform a simulation. Among the 19 included software, eight were considered intuitive and user-friendly (Tucuxi, PrecisePK, ID-ODS, RxStudio, MwPharm, iDose, DoseMe, and InsightRX Nova). All of these software were web-based, and all, except for ID-ODS, had the capability of EHR integration, which enhanced the user experience. They also shared a modern interface where the path to run a simulation was easily identifiable. Four other software (NextDose, BestDose, TDMx, and RxKinetics) were also considered relatively easy to use, although their interfaces were less user-friendly and the workflow was less assisted compared to the previous group. PKS and JPKD were not considered user-friendly due to their outdated interfaces and lack of smooth flow through the screens. NONMEN deserves special mention as its use requires a high level of expertise not only in pharmacokinetics and pharmacometrics but also in programming, making it less accessible in clinical settings. Lastly, Kinetidex, Autokinetics, CAPCIL, and TCIworks were not accessible to the investigators, so their ease of use could not be evaluated.”

The corrections have been made to Section 4 (Paragraph 8), which are a consequence of the inclusion of ID-ODS as a separate software in the review:

“Indeed, the quality of the software, user support services, supported drugs and population models, as well as output generation, are crucial considerations for selecting a MIPD software. The study by Kantasiripitak et al. [1] positioned DoseMeRx, InsightRX Nova, and MwPharm as excellent options for dose optimization in clinical practice. Similarly, the review by Fuchs et al. [7] identified MwPharm, MM-USC*PACK (currently BestDose), and TCIWorks as the most complete programs. In our opinion, software that includes population PK models for special populations, such as obese patients, critically ill patients, neonates, or patients on dialysis, provides added benefits for professionals who frequently treat patients with more complex requirements. PrecisePK and ID-ODS stand out as the most complete tool in this regard, as it includes models for each of the mentioned target populations. However, ID-ODS is only focused on antimicrobial and antifungal agents, whereas PrecisePK includes a wide range of drug types. Additionally, DoseMeRx, MwPharm, and RxStudio offer the ability to predict optimal dosing regimens for various special populations. Apart from special populations, DoseMeRx, InsightRX Nova, and MwPharm provide the largest collection of population PK models by default. These aspects facilitate the implementation of such software in clinical practice.”

The corrections were made in Table 1, which include the information related to ID-ODS software. The corrected Table 1 appears below.

The authors state that the scientific conclusions are unaffected. This correction was approved by the Academic Editor. The original publication has also been updated.

## Figures and Tables

**Table 1:** Descriptive characteristics of the MIPD software.

	Autokinetics	BESTDOSE	CAPCIL	DoseMeRx	iDose	InsightRX Nova	JPKD	Kinetidex	MwPharm	ID-ODS
**General characteristics**										
Developer/ promoter	Paul Elbers, Rob Bosman. Departments of Intensive Care Medicine of Amsterdam UMC	R.W. Jelliffe. Laboratory of Applied Pharmaco-kinetics, University of Southern California, LA. Non-company owners	Company (SIMKIN Inc.), USA	Robert McLeay. DoseMeRx^®^ (Tabula Rasa Healthcare Company)	Company (Projections Research Inc. Baysient^®^)	Sirj Goswami, Ron Keizer and Ranvir Mangat. Company(Insight Rx Inc.)	College of Pharmacy, Kaohsiung Medical University (Taiwan)	Thomson Reuters Corp. (merge between Simkin and Micromedex)	Johannes H. Proost Department of Pharmacology and Therapeutics, University Centre for Pharmacy, Groningen. Company (Mediware a.s.)	Andras Farkas. Company (Optimum Dosing Strategies)
Year of creation	2018	2018	N/A	2014	N/A	2015	2006	2001	1987	2013
Geographical location	Netherlands	USA	USA	USA	USA	USA	Taiwan	USA	Netherlands	USA
Languages available	English	English *	N/A	English	English	English, Dutch, German, French. Possibility of development	English *	English	English, Spanish, Dutch, German, Korean, Czech, Portuguese, Japanese, Chinese	English
Last update	N/A	Version 2.4.3 Previous version 1973: MM-USC*Pack [7]	N/A	N/A	November 2022	The platform is updated regularly (once a month). Previous version: InsightRX software [14]	2008 version 3.0	2009 Version 9.0	September 2022 Version 2.2.0	2023
**Access options**										
Testing the software	Yes	Yes	N/A	Yes, a 14-day trial can be generated	Yes	Yes	N/A	N/A	Yes	Yes
Subscription	Paid access	Paid access	Unknown	Paid access	Paid access	Paid access	Free access	Paid access	Paid access	Paid or free access
Platforms	Desktop and web-based: www.autokinetics.eu	Web-based www.lapk.org/bestdose.php	Desktop	Mobile application and web-based: https://doseme-rx.com/	Web-based: www.baysient.net/idose-product/	Web-based: www.insight-rx.com/	Web-based: pkpd.kmu.edu.tw/jpkd/	Desktop	Desktop, mobile application, and web-based: www.mediware.cz/	Web-based: https://www.optimum-dosing-strategies.org/id-ods/
**Technical characteristics**										
Pharmacokinetic analysis type available	Bayesian and non-Bayesian	Bayesian (non-parametric approach)	Bayesian and non-Bayesian	Bayesian (parametric approach)	Bayesian	Bayesian	Bayesian	Bayesian	Bayesian (parametric approach)	Bayesian (parametric approach)
Inclusion/not by default of population data	Yes	Yes, it offers a collection of population PK models	N/A	Yes, it offers a collection of population PK models	Yes	Yes, it offers a collection of population PK models	N/A	Yes	Yes, it offers a collection of population PK models	Yes, it offers a collection of population PK models
Inclusion of drugs and populations PK models	No	No	N/A	Yes, upon request	Yes, upon request	Yes, upon request	Yes, by the user	No	Yes, by the user or upon request	Yes, upon request or using free- style simulation routines by the user
Issuance of reports, creation of graphs	Reports cannot be generated. Graphical representations can be generated (not exported)	Reports cannot be generated.	Reports and graphical representations can be generated	Reports and graphical representations can be generated	Graphical representations can be generated	Reports and graphical representations can be generated	Reports can be generated	Reports and graphical representations can be generated	Reports and graphical representations can be generated	Reports and graphical representations can be generated
Integration into EHR/EMR	Yes. It can be integrated	N/A	N/A	Yes. It can be integrated	N/A	Yes. It can be integrated	N/A	N/A	Yes. It can be integrated	No
**Clinical applicability**										
Drug class	Antibiotics	Antibiotics, digoxin	Antibiotics, digoxin, lidocaine, quinidine, theophylline	Antibiotics, anticoagulants, anticonvulsants antifungals, antineoplastics, antithrombotics, immunosuppressants (drugs for transplants), digoxin, warfarin	Immunosuppressants (biological agents)	Antibiotics, anticoagulants (oral, factors), antifungals, antineoplastics, antipsychotics, antithrombotics, digoxin, immunosuppressants (both drugs for transplants and biological agents), methadone	Anticonvulsants, antiepileptics, antivirals, digoxin, immunosuppressants (drugs for transplants), lithium, theophylline, warfarin	Antibiotics, antiepileptic, digoxin, theophylline, warfarin	Antibiotics, antiepileptics, antihypertensives, antivirals, immunosuppressants (biological agents), digoxin, warfarin	Antibiotics and antifungals
Target population †	N/A	N/A	Adults, neonates, and pediatrics	Adults, neonates, and pediatrics. Hemodialysis and obese	Adults and pediatrics	Adults, neonates, and pediatrics	N/A	N/A	Adults, neonates, and pediatrics. Critically ill patients and hemodialysis	Adults, neonates, and pediatrics. Critically illpatients, obese, renal replacement surgical, obese, burned and cystic fibrosis
	NextDose	NONMEM	PrecisePK	PKS	RxKinetics	RxStudio	TCIWorks	TUCUXI	TDMx	
**General characteristics**										
Developer/ promoter	Sam Holford Nick Holford. University of Auckland Non-company owned	Project Group at the University of California, San Francisco Company (NONMEM^®^)	Philip Anderson, Anjum Gupta. Company (Healthware Inc.)	Company (Abbott Laboratories, Diagnostic)	School of Pharmacy and Health Profession, Creighton University	Ajay Gopal, Gergely Daroczi. Company (Rx Studio Inc.)	University of Queensland (Australia) and University of Otago (New Zealand)	Yann Thoma. School of management and engineering of Vaud and the University Hospital of Lausanne	Sebastian Wicha. Institute of Pharmacy, University of Hamburg	
Year of creation	2012	1979	1986	1991	1984	2020	2011	2013	2015	
Geographical location	New Zealand	USA	USA	USA	USA	USA/Hungary	New Zealand and Australia	Switzerland	Germany	
Languages available	English	English	English, Spanish, Korean, Chinese, Thai	English	English	English, Spanish, Portuguese, French, Hungarian, and Simplified Chinese	English *	English	German, English	
Last update	September 2022	NONMEM 7.5.1 February 2022	September 2022 Previous version: T.D.M.S.	N/A	November 2021	February 2023. The platform is updated regularly.Previous version: ID-ODS	N/A	November 2022	February 2023	
**Access options**										
Testing the software	No	N/A	Yes, a 30-day trial can be generated	N/A	Yes, a 60-day trial version can be downloaded	Yes	N/A	Yes	Yes	
Subscription	Paid or free access	Paid access	Paid access	Paid access	Paid access	Paid access. Free individual access for empirical simulations	Free access	Free access	Free access	
Platforms	Web-based: nextdose.org/	Desktop	Desktop and web-based: precisepk.com/	Desktop	Web-based: www.rxkinetics.com/	Desktop, mobile application, web-based: rx.studio/	Desktop	Desktop	Web-based: www.tdmx.eu/	
**Technical characteristics**										
Pharmacokinetic analysis type available	Bayesian	Bayesian	Bayesian	Bayesian and non-Bayesian	Bayesian	Bayesian (parametric approach)	Bayesian (parametric approach)	Bayesian	Bayesian	
Inclusion/not by default of population data	Yes, it offers a collection of population PK models	No. Users may define model for any drug or target population	Yes, it offers a collection of population PK models	Yes, by the user	Yes	Yes, it offers a collection of population PK models	Yes	Yes, it offers a collection of population PK models	Yes, it offers a collection of population PK models	
Inclusion of drugs and populations PK models	No	Yes, by the user	Yes, upon request	Yes, generation of new drugs in different populations	Yes, upon request	Yes, upon request	Yes, by the user	Yes, by the user and supported by the developer	Only new population (not new drugs), implemented by the user	
Issuance of reports, creation of graphs	Reports and graphical representations can be generated	Reports can be generated	Reports can be generated	Reports and graphical representations can be generated	Reports and graphical representations can be generated	Reports and graphical representations can be generated	Reports can be generated	Reports and graphical representations can be generated	Reports cannot be generated. Graphical representations can be generated (not exported)	
Integration into EHR/EMR	No	N/A	Yes. It can be integrated	N/A	N/A	Yes. It can be integrated	N/A	Yes. It can be integrated	No	
**Clinical applicability**										
Drug class	Antibiotics, anticoagulants, antifungals, antineoplastics, antirheumatics, antivirals, immunosuppressants (drugs for transplants), psychostimulants, warfarin	N/A (selected by the user)	Antiarrhythmicantiasthmatics, antibiotics, antiepileptics, antifungals, antineoplastics, antipsychotics, immunosuppressants (drugs for transplants)	Antibiotics, digoxin, methotrexate, phenytoin, theophylline	Antibiotics, digoxin	Antibiotics, antifungals, anticonvulsants, biological agents, immunosuppressants (drugs for transplants), methotrexate, opioids	Antibiotics, immunosuppressants (drugs for transplants), theophylline, warfarin	Antibiotics, anticoagulants, antivirals, antineoplastics, immunosuppressants (drugs for transplants), kinase inhibitors	Antibiotics, haemostatics (factors), immunosuppressants (biological agents)	
Target population †	Adults, neonates, and pediatrics	N/A	Adults, neonates, pediatrics. Critically ill patients, obese, renal impairment and hemodialysis	Adults and pediatrics.Critically ill patients	Adults and pediatrics	Adults, neonates, and pediatrics. Hemodialysis and hematology patients	Adults and pediatrics	Adults, neonates, and pediatrics	Adults, neonates, and pediatrics	

* Availability in other languages unknown. † At least one model per target population.

## References

[1] Del Valle-Moreno P., Suarez-Casillas P., Mejías-Trueba M., Ciudad-Gutiérrez P., Guisado-Gil A.B., Gil-Navarro M.V., Herrera-Hidalgo L. (2023). Model-Informed Precision Dosing Software Tools for Dosage Regimen Individualization: A Scoping Review. Pharmaceutics.

