# Supplementary material for: Model-Informed Precision Dosing Software Tools for Dosage Regimen Individualization: A Scoping Review
Source: Pharmaceutics. 2023 Jul 1;15(7):1859. doi: 10.3390/pharmaceutics15071859 (PMC10386689; doi:10.3390/pharmaceutics15071859)
Supplement: Supplementary file 1 [file pharmaceutics-15-01859-s001.zip › pharmaceutics-2437986-supplementary.pdf]

Supplementary Figure S1. Preferred Reporting Items for Systematic reviews and Meta-Analyses extension for Scoping Reviews (PRISMA-ScR) Checklist [9].

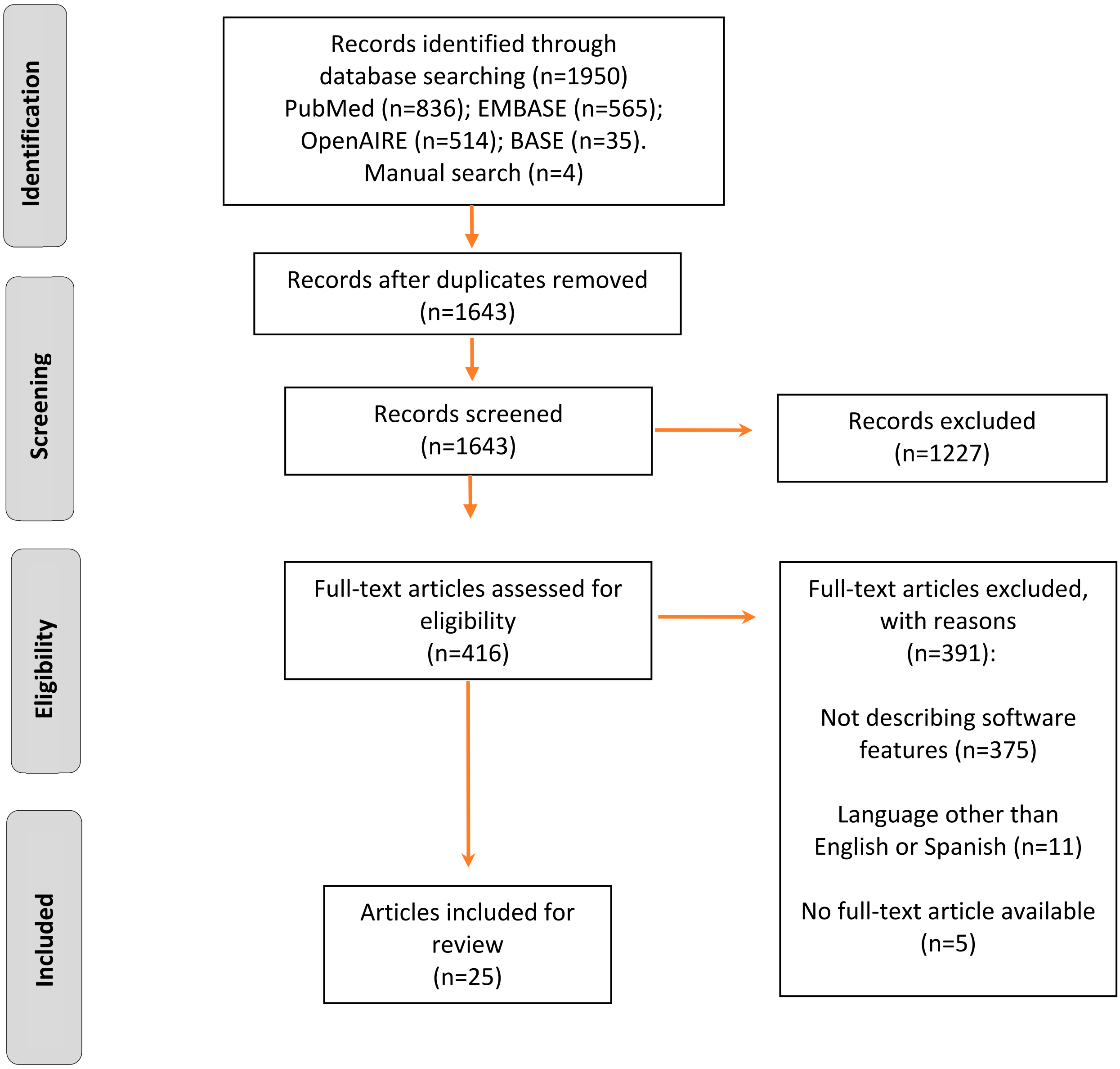

Supplementary Table S1. Search strategy

**Databases**	**Search strategy**
MEDLINE (PubMed)	(“therapeutic drug monitoring” OR “Drug Dosage Calculations”) AND (“software” OR “Drug Therapy, Computer-Assisted” [MeSH Terms] OR “Population pharmacokinetics”)
Embase	(‘therapeutic drug monitoring’ OR ‘drug dosage calculations’) AND (‘software’/exp OR ‘computer assisted drug therapy’/exp OR ‘population pharmacokinetics’/exp)
OpenAire	(“therapeutic drug monitoring” AND “software”)
BASE	(“therapeutic drug monitoring”, “drug therapy software”, “drug dosage software” AND “pharmacokinetic software”)

Supplementary Table S2. Summary of the articles included in this review

	Author, year	Country	Objective	Software described
1	Hatton et al. 1984 [17]	USA	Compare the accuracy of microcomputer-predicted versus trough serum concentrations during long-term tobramycin therapy	CAPCIL (SIMKIN)
2	Deci et al. 1985 [18]	USA	Assess the ability of a computer program Simulated Kinetics (SIMKIN) to predict serum theophylline concentrations in ambulatory patients receiving oral theophylline	CAPCIL (SIMKIN)
3	Leal et al. 1991 [19]	Belgium	Describe a software developed to optimize aminoglycosides monitoring, responding to the demands of most clinical daily situations	PHAR-MONITOR
4	Messori et al. 1992 [6]	Italy	Present a microcomputer program which analyses multiple-dose pharmacokinetic curves using either a least-squares nonlinear analysis or a Bayesian fit	PKRD
5	Proost et al. 1992 [20]	Netherlands	Describe the main features of MW/PHARM and exemplify with gentamicin and phenytoin	MWPHARM
6	Gill et al. 1992 [21]	USA	Report the use of a new method to determine patient population pharmacokinetic parameters compared to a more traditional approach	NPEM (USC*PACK)
7	McMichael et al. 1993 [22]	USA	Validate an intelligent dosing system (IDS) used to guide all doses to prospectively achieve the target concentration range specified in autoimmune disorders	IDS
8	Ismail et al. 1993 [23]	Malaysia	Estimate individual and population pharmacokinetics for carbamazepine in Malaysian epileptic patients using the OPT computer program	OPT
9	Lacarelle et al. 1994 [24]	France	Validate a new software package in routine clinical practice for amikacin and theophylline	PKS
10	Jerling, 1996 [25]	Sweden	Demonstrate how the population approach can be used to evaluate different kinetic and dynamic aspects of a drug and to give some suggestions for how to utilize the results in clinical practice	NPML
11	Falcao et al.	Portugal	Study the kinetic behaviour of theophylline administered concomitantly with methylprednisolone and auranofin, using PKS	PKS and CAPCIL

	2000 [26]		and CAPCIL for the kinetic analysis	
12	Wright et al. 2011 [27]	New Zealand	Develop a Bayesian dose individualisation tool for warfarin and incorporate it into the freely available software TCIWorks for use in the clinic	TCIWorks
13	Neely et al. 2012 [28]	USA	Describe “Pmetrics”, a newly software package and test the theoretical advantage of the nonparametric approach to accurately characterize subpopulations and outliers by estimating population and individual pharmacokinetic parameters in a simulated data set with the parametric IT2B and nonparametric NPAG algorithms in Pmetrics	NPAG and IT2B
14	Fuchs et al. 2013 [7]	Switzerland	Assess and compare computer tools designed to support TDM clinical activities	JPKD, TDM for R, RxKinetics (Antibiotic Kinetics, APK and Kinetics), Kinetidex, T.D.M.S. 2000, DataKineticsTM, RADKinetics, MM-USC*PACK, TCIWorks, MwPharm
15	Felton et al. 2014 [29]	UK	Establish a population PK model for piperacillin in critically ill patients and analyze the performance of the model in the dose optimization software program BestDose	BestDose
16	Dubovitska et al. 2017 [2]	Switzerland	Present TUCUXI – an intelligent system for TDM and discuss ethical issues related to the use of an automated decision support system in clinical practice	TUCUXI
17	Owens et al. 2018 [14]	USA	Explain advances in technology that allow clinicians to apply PK-PD to optimize the agents and dosing regimens selected for the treatment of hospitalized patients with infection	PK-PD Compass, TDMx, DoseMeRx and InsightRx
18	Nugroho et al. 2019 [30]	Indonesia	Simulate the capability of Monolix, NONMEM, and WinBUGS-PKBUGS to analyze very sparse Cp-time data after an intravenous bolus drug administration and to estimate the minimum number of Cp-time data required for an adequate analysis	Monolix, NONMEM, and WinBUGS-PKBUGS
19	Okour, 2020 [31]	USA	Present DosePredict, a Shiny-based graphical user interface software that can be used for	DosePredict

			the conduct of dose predictions	
20	Kantasiripitak et al. 2020 [1]	Belgium	Identifying the requirements for and evaluating the performance of the currently available model-informed precision dosing software tools	DoseMeRx, InsightRX Nova, MwPharm++, PrecisePK, Kinetics, BestDose, ID-ODS, NextDose, Tucuxi, Autokinetics, TDMx
21	Porubän et al. 2020 [32]	Czech Republic	Compare the usefulness of DOS and Windows version (WIN) of the MwPharm, and their prediction quality in TDM of digoxin	MwPharm
22	Ryan et al. 2021 [33]	Australia	Evaluate amikacin prescribing and TDM practices, and determine the suitability of the amikacin model incorporated into the DoseMeRx® software as a replacement for the previously available software (Abbottbase®)	Abbottbase, DoseMeRx
23	Dave et al. 2021 [34]	Germany	Compare measured Infliximab concentrations in the laboratory with values predicted by iDose dashboard system and report its efficacy in managing patients not responding to conventional dosing schedule	iDose
24	Sturkenboom et al. 2021 [35]	Australia	Provide an update on the pharmacokinetics and pharmacodynamics of anti-tuberculosis drugs and to show how population pharmacokinetics and Bayesian dose adjustment can be used to optimize treatment.	NONMEM, Pmetrics, ADAPT, MwPharm, Monolix, Phoenix, and NPEM2
25	Heitzmann et al. 2022 [36]	France	Implement and compare two pharmacometric tools for daptomycin TDMand precision dosing	BestDose and TUCUXI

Supplementary Table S3. Software tools for modelling or research

Function/use	Software tool
Modelling	ADAPT, IT2B, NPAG, NPML, Monolix, PhoenixNLME, WinBUGS-PKBUGS, WinNonlin
Research	DosePredict
